# A qualitative study of perception related to risky driving behavior in Debre Markos City, North West Ethiopia, 2021

**DOI:** 10.1186/s12889-023-15862-x

**Published:** 2023-05-26

**Authors:** Elyas Melaku Mazengia, Ayenew Kassie, Amare Zewdie, Getu Debalkie Demissie

**Affiliations:** 1 Department of Public Health, College of Health Science, Debre Markos University, Debre Markos, Ethiopia; 2 Department of Health Promotion and Health Behavior, College of Medicine and Health Science, University of Gondar, Gondar, Ethiopia; 3 Department of Public Health, College of Medicine and Health Science, Wolkite University, Wolkite, Ethiopia

**Keywords:** Perception, Risky driving behavior, Driver, Qualitative, Ethiopia

## Abstract

**Background:**

About 1.35 million deaths and around 50 million injuries are attributed to road traffic crashes every year in the world. In Ethiopia, road traffic crashes contributed to a fatality rate of 37 per 100,000 populations per year, and 83% of traffic crashes were attributed to risky driving behavior. This study aimed to explore perceptions related to risky driving behavior among public transport vehicle drivers in Debre Markos City, North West Ethiopia, in 2021.

**Methods:**

A generic qualitative study was conducted from August 05– September 15, 2021. A total of 17 participants (10 drivers, 4 drivers’ training school instructors, and 3 traffic police officers) were selected by a purposive heterogeneous sampling technique. An open-ended interview guide was used during the interview, and all interviews were audio recorded. Data collected in the local language was transcribed verbatim and translated into English. The ATLAS-TI version 7.5 software was used to code the data, and finally, thematic analysis was done.

**Result:**

Four themes were identified. The first theme was “transport safety rule and enforcement problem,” which includes gaps in the transport safety rule itself and gaps in the implementation of the rule. The second theme was “Drivers’ training curriculum and application gaps,” which focuses on gaps in the training curriculum and its application during recruitment, training, and examination of trainees. The third theme was “technical and financial problems”. This theme includes problems related to the vehicles’ technical issues and the appropriateness of transport tariffs. The final theme was “passenger and vehicle owners’ related problems”. This theme is about the influence of passengers’ and vehicle owners’ practices on drivers’ risky driving behavior.

**Conclusion:**

Revising transport safety rules and strictly following the implementation of the drivers’ training curriculum and transport safety rules should be given due attention. In addition, behavior change communications tailored to drivers and vehicle owners could be beneficial in reducing risky driving behaviors.

**Supplementary Information:**

The online version contains supplementary material available at 10.1186/s12889-023-15862-x.

## Introduction

The World Health Organization’s 2021 report showed that road traffic crashes (RTCs) are a pervasive public health problem in the world, with an estimated annual death rate of 1.3 million people and up to 50 million disabilities [1]. It is the leading cause of death in the age group of 5–29 years old and the 8th leading cause of death for all age groups in the world [2]. Around 93% of deaths were reported in low- and middle-income countries. However, these countries own 60% of the vehicles in the world [1]. Africa carries the highest burden of RTCs [2], with a road traffic crash rate of 65.2 per 100,000 population and a fatality rate of 16.6 per 100,000 population [3]. RTCs were the second most common form of injury and accident in Ethiopia, which contributed to around 43% of fatalities, with a 163 per 100,000 population per year rate of accidents and a fatality rate of 37 per 100,000 population per year [4]. A study done in Ethiopia using 11 years of data (2007/08–2017/18) showed that, on average, 26,507 traffic crashes were reported, with an annual death toll of 3,345 people. Furthermore, over the 11-year study period, the annual rates of fatalities and severe injuries increased by 9.28% and 10.76%, respectively. RTCs also caused an estimated national loss of 33.6 billion Ethiopian birrs in 11 successive years, or 3.3 billion birrs per year [5].

Risky driving behaviors (RDBs) such as speeding, improper overtaking, reckless driving, fatigue, drunk driving, drugs, and cell phone use while driving were responsible for 75% and 83% of RTCs in Africa and Ethiopia, respectively [6]. A study done in the Amhara Regional National State of Ethiopia also reported that around 83.3% of the RTCs in that region were attributable to RDBs like speeding, driving in the left lane, driving too close to other vehicles, and failing to give priority to both vehicles and pedestrians [7]. According to the East Gojjam Zone, Amhara Regional National State Traffic and Road Safety Offices’ first six-month report of the 2020/21 fiscal year, a total of 218 RTCs were recorded, with the deaths of 200 people and heavy and minor injuries of 145 and 160 people, respectively. The death rate showed a 47% increment as compared to the previous year’s similar time report. Of the total crashes, almost half (48.2%) occurred in public transport vehicles, of which 83.8% were in vehicles with 12–45 seats. Speeding was the cause of 41% of the total accidents in the zone [8]. Another study revealed that most RTCs (35%) occurred among passenger vehicle occupants [4]. Thus, our study focused on passenger vehicle drivers.

Even though those aforementioned behavioral factors contributed to the highest proportion of RTCs in Ethiopia, most studies mainly focused on determining the magnitude of RTCs, and there is a paucity of evidence exploring drivers’ behavior at the national level in general and in the study area in particular. This study aimed to explore perceptions related to RDBs among public transport vehicle drivers in Debre Markos City, 2021, which will serve as input to design and implement efficient RTC prevention projects and programs.

## Methods

Study designA series of interviews were conducted following a generic qualitative approach. A generic approach [9, 10] is not guided by one of the known qualitative methodologies such as phenomenology, ethnography, or grounded theory. Thus, this approach allows for greater flexibility in describing events in their natural setting without having a specific theoretical or philosophical base [11].

### Study setting and period

The study was conducted from August 5–September 15, 2021, in Debre Markos City, Amhara National Regional State, Ethiopia. Debre Markos is located 295 km away from Addis Ababa, the capital city of Ethiopia, and 255 km away from Bahirdar, the capital city of the Amhara Regional National State. It is the administrative center of the East Gojjam Zone. The zone has 17 rural districts and 5 city administrations, with a total population of 2,255,288 in 14,004 km^2^ [12]. In this city, there is one public transport vehicle station that serves as the departure and destination for many people traveling in and out of the city. There were a total of 3,880 public transport cars in the East Gojjam zone, of which 1,406 had 12–45 seats. All these cars were privately owned, and the drivers could be the owners of the car or employees. Employed drivers work for a monthly salary, and all drivers were male. Their salary depends on the daily net profit that they give to the owner of the car. In this zone, approximately 73 traffic policies were in place to ensure transportation safety [13]. This zone had 10 hospitals, 104 health centers, and 485 health posts with 3,599 health professionals [14].

### Study participant selection

This study investigated the perception behind RDBs by focusing on public transport vehicle drivers. The reason to focus on public transport vehicle drivers was that the magnitude of crashes and the devastating outcomes follow accidents in passenger vehicles as compared with freight transport vehicle drivers. Different scholars recommend different sizes of research participants depending on the design of the study, the heterogeneity of the population, and the richness of the information. For instance, Lincoln and Guba recommend 12–20 participants for interview-based studies [15]. By considering these points, a total of 19 participants (12 passenger vehicle drivers who had worked for more than one month, 4 driver training school instructors, and 3 traffic police officers) were initially selected using a heterogeneous purposive sampling technique. This sampling technique is used to get a more diversified and representative sample [16] based on age, experience, marital status, and educational status. To get participants, the data collector initially contacted the coordinator at the bus station and then directly communicated with drivers in the bus station while they were waiting for their turn to load passengers. After describing the aim of the study, the interviewer received the phone numbers of interested participants for further communication. A total of 19 drivers who showed interest in participating in the research were further contacted by telephone to decide their preferred place and time for an interview. Interviews were stopped after interviewing 17 participants after reaching a level of data saturation, the time at which no new data is generated from continuing interviews [17]. The principal investigator, who has taken advanced qualitative research methods and has experience collecting qualitative data, conducted in-depth interviews with drivers, traffic police officers, and driving school instructors. An open-ended interview guide specific to the group of participants (drivers, traffic police officers, and driving school instructors) was prepared by reviewing different literature. These interview guides were evaluated by three health promotion experts before being used for the interview. In addition to voice recording, note-taking was used to collect data. To optimize the quality of the data, different time- and place-related contexts were taken into consideration. For instance, drivers were interviewed at a time when they were free from any work in clean and safe areas. All interviews were also done in the morning to decrease the effect of work-related fatigue on the quality of the data. All key informants were interviewed at their working site in the absence of other people other than the interviewer, to give them more freedom to express their ideas and decrease socially desirable answers. The interviews lasted between 25 and 43 min.

### Trustworthiness and rigor

Qualitative research, like quantitative research, has criteria to assure the quality of the research method and data. Through the application of different techniques, the trustworthiness and rigor of qualitative findings (credibility, dependability, transferability, and confirmability) were assured [18]. According to the positivist/post-positivist paradigm, the criteria for assessing trustworthiness are credibility, transferability, dependability, and confirmability [19]. The following measures were taken to ensure the credibility of the research: Persistent engagement with the interviewee was considered to establish rapport and trust between the interviewer and the interviewee. It was also helpful to specify a preferred day for the interview. The aims of the research were explained to each participant to prevent socially desirable responses. Participant triangulation was made to compare and verify a response given with other responses and previously known facts. A critically evaluated interview guide that could address the aim of the study was used to collect data. The number and sociodemographic profile of participants involved in the interview, methods used to select them and collect data, and time, place, and duration of data collection were described to optimize the transferability of this finding. Even though actions taken to increase the credibility of this result may also increase dependability, the research design, method of participant selection, data collection, and analysis techniques used were explained to help readers and other researchers judge whether this research followed appropriate scientific procedure and whether the findings were reliable or not. An audit trail that includes voice recordings of interviewed participants, translated Word documents, codebooks, and software outputs with codes was prepared to maximize the confirmability of this result.

### Data analysis

The data were verbatim transcribed in Amharic (the local language) and then translated back to English. The researchers translated the data, so no software was used to translate the transcript. After reading and re-reading the translated document, coding was done. Thematic analysis with the codebook approach was used. Thematic analysis is the most commonly used and flexible method of analyzing qualitative data by identifying and describing patterns and interpreting them during the process of coding and constructing themes [20]. The codebook approach is between coding reliability and the reflexive approach, which accepts subjectivity and follows a structured approach to the coding process without testing reliability [21]. after the authors (researchers) were familiarized with the transcript, the initial codes were generated by the three researchers. Peer debriefs were done by the three researchers. After the codes were generated, the initial themes were generated, and detailed notes were documented about the connection between codes and themes. A review of the themes and sub-themes was discussed. The team reached the point of naming the themes. For simplicity and common consensus on the codes and themes, a codebook was prepared. The codebook contained the possible codes, themes, and meanings of the codes and themes. Then the three researchers independently coded the transcript. The themes emerged from the codes. The coding process was done on the ATLAS TI-7.5 version of the software.

## Findings

### Socio-demographic characteristics of participants

A total of 17 participants were involved, of which 10 were drivers (6 minibus drivers (12–16 seats) and 4 drivers (24–44 seats)). Four driver training school instructors and three traffic police officers were interviewed. A greater number of participants (7 (41.2%)) were within the age group of 20–30 years old, and 14 (82.4%) were males. Furthermore, nearly one-third (29.4%) of the participants were educated to the level of primary school, and they had 3–5 years of work experience (Table 1).

**Table 1:** Socio-demographic characteristics of interview participants to explore perception related to RDB

Variables	Category	Frequency (%)
Age	20–30	7 (41.2)
	31–40	4 (23.5)
	41–50	4 (23.5)
	51–60	2 (11.8)
Sex	Male	14 (82.4)
	Female	3 (17.6)
Educational status	Primary school	5 (29.4)
	Secondary and preparatory school	9 (53)
	College and above	3 (17.6)
Marital status	Single	7 (41.2)
	Married	8 (47)
	Divorced	2 (11.8)
Occupation	Driver	10 (58.8)
	Driver training school instructor	4 (23.5)
	Traffic police	3 (17.7)
Work experience	0–2 years	3 (17.7)
	3–5 years	5 (29.4)
	5–10 years	5 (29.4)
	> 10 years	4 (11.8)

### Perceptions related to risky driving behavior

Four themes emerged regarding the driving force of drivers toward RDBs. These are transportation safety rules and enforcement issues; driver education program and application gaps; vehicle ownership and passenger issues; and issues associated with financial and technical issues.

### Theme 1: driver training curriculum and issues with its application

Ethiopian drivers’ training curriculum includes classroom teaching supported by lab and field practical sessions. This theme includes two basic components. The first is gaps in the curriculum, and the second is issues with its implementation. The curriculum implementation gap occurs in three main phases, starting from entry to training school, the processes of training, and the exam delivery system.

The training curriculum was mentioned as a contributing issue to drivers’ engagement in RDBs. Problems identified in the training curriculum include a shortage of time to deliver both theoretical and practical sessions and the delivery of higher-level licenses in one phase. Furthermore, the controversial contents of the national driving training manual and computer-based driving license qualification exam were also identified as contributing to drivers’ engagement in RDBs.

The majority of participants raised the issue of time constraints for delivering both theoretical and practical sessions. Furthermore, many participants also mentioned that the current way of delivering higher-level driving licenses in one phase is one driving force for drivers’ engagement in RDBs.

“*We became drivers by working as assistants and taking the license every year, but now they simply come out of their homes and take the license, and they cause accidents.“ At least licenses should be delivered every year or every six months in a level-based manner to improve drivers’ competence.*“ (Driver)

*”The training time of two months is too short to deliver both theoretical and practical sessions. So it should be extended at least for six months, and it would be great if the training was delivered at technical and vocational colleges.“* (Driver training school instructor)

Even though the majority of participants agreed that the training document was appropriate, some participants explained that it contained terms and concepts that were directly transcribed from English, which is difficult to understand.

Two major problems related to the question bank were also mentioned by most participants. The first problem was the content of the question bank, which included questions without correct answers. The second problem was the inability of the questions to measure trainees’ knowledge equally and appropriately.

As most participants agreed, drivers who are getting licenses usually don’t fulfill legal entry criteria like age > 24 years and an academic certificate, which requires a grade ten certificate. In addition, participants also mentioned that those who are joining the driving profession don’t understand how much the profession is respected and crucial.

They frequently mentioned that students who attend driving schools simply join the school because they failed in other fields of work and for the sake of temporary gain. They also described how students who registered to take training didn’t attend classes properly; some students also fully ignored theoretical sessions and focused only on practical sessions. They also indicated ways in which driving licenses were being delivered for those who didn’t attend any driving-related sessions. Under training-related issues, how exam results are changed and those who do not perform well obtain driving licenses were also major sources of concern for many participants.

*“Usually, trainees who attend these schools are individuals who fail to score a passing mark and attend preparatory schools; they take driving as the only work to do for a living. Most students prefer practical sessions and fully ignore theoretical ones, which is the key to changing their behavior. There is also no strict control over those students who miss the theoretical sessions.“ (Driver training instructor)*

”*They don’t fulfill academic requirements; simply, institutions adjust this. Underage people can get the license. Age can be corrected on ID cards before registration.“ (Driver).*

*“Nothing prohibits you if you have money. Even I can buy your master’s and your degree.“ (Driver training instructor)*

### Theme 2: transport safety law and gaps in implementation

Under this theme, issues related to traffic safety rules, regulations, and enforcement concerns are included. Some participants described how the traffic safety law by itself is contributing more to drivers’ engagement in RDBs because the road safety law is frequently changed, different across different administrative areas within a country or even in the region, and has an imbalance in violations and corrective measures taken.

“*There has been a wide gap in the transport safety law since the 2002 Ethiopian Calendar (2010 GC). After I was assigned to this job, the law was changed four to five times; before you read and understand one law, another may come, and it is complicated. Now, even though it is not expected to say this, the law seems prepared to benefit drivers who engage in RDB. " (Traffic police officer)*

The enforcement problem of road safety rules and regulations mentioned by participants includes unequal treatment of drivers committing equal violations, complete failure to control and take proper measures for violations, and taking extreme measures for minor violations. Many participants explained that the unequal treatment of drivers by transport office workers and traffic police staff opened the door to corruption and the advancement of RDBs. They also described that in the case of extreme measures taken for minor violations, drivers might become emotional and aggressive while driving, which could be followed by speeding and overloading passengers. Similarly, they indicated that when there is a complete failure to implement safety laws, most drivers exercise RDBs.

“*In the case of our country, a person who killed 10 people may go out of prison through different relationships, including corruption; on the contrary, if a driver is poor, and has no influential relatives, he may get imprisoned for pushing or causing minimal injury to a person.“ (Driver*)

*”For example, when I drove in Addis Ababa (the capital city of Ethiopia), I used to fasten the belt and unfasten it again by considering it like our local area; I have been accused and punished two times, but here there is no traffic light and no one controls the seat belt; it is common to see faults; no one applies the turn light and signals while turning” (Driver).*

The majority of participants also believed that the violation of one safety rule could cause drivers to engage in additional RDBs.

They mentioned as an example that drivers who load too many passengers may drive fast to pass before traffic police arrive on the road, may improperly overtake to skip traffic police, or may make a phone call while driving to get information about where traffic police are located.

They also added that a driver who is addicted to drugs may overload passengers to maximize profits and fulfill substance needs, and that person may also drive with speed and improperly overtake to arrive at the place early where addictive substances are found.

*“Most of the time, accidents occur when drivers attempt to pass traffic police by overtaking other vehicles when the driver doesn’t catch an exit card or overload passengers” (Driver).*

*“When a driver chews chat while driving, he may drive with speed or overload passengers to maximize his profit for his addiction, because a person who chews in the daytime must drink alcohol to get asleep at night; they need more time and money to do all these things... I have friends who do this.“ (Driver)*

### Theme 3: passengers’ and vehicle owners’ behavior

Participants mentioned that passengers contributed to drivers’ engagement in RDBs. Some participants warned that when females and drivers’ close friends sit in the front seats, drivers might exceed the speed limit, overtake in dangerous situations to entice that person, or be admired by some front-line personnel. Some drivers also explained that passengers make them drive fast and that if they do not, a passenger could take action, such as taking other cars.

*“Giving females the front seat next to drivers and driving while talking, overacting, or fighting with passengers for a small sum of money can result in an accident” (driver).*

“*If you drive at 80 km/hr, passengers may come from the back and shout at you, saying are we traveling by donkey? Some passengers get depressed when other cars overtake them, and once upon a time, my car had a defect in its speed, then passengers ordered me to stop, and they went out and took another car” (driver)*.

This study indicated the influence of car owners on the behavior of drivers. Here, the most commonly described issue related to owners of cars is employing drivers based on social and blood relations, regardless of their driving behavior, skill, and experience. Some participants also indicated that the way the owner of the car buys the car through credit is the main problem that influences the driver’s behavior. They explained that since such owners need more income to cover their credit, they may push themselves or their drivers to transport passengers beyond the car’s carrying capacity, to drive with speed, and to overtake improperly.

“Now *drivers are being employed by considering blood relationships, and someone from the rural area may directly come and take a license without properly attending classes and starting routine driving work at the time they cause an accident” (Driver).*

*“Nowadays, most vehicle owners buy cars on credit, and these owners put pressure on themselves or their drivers to maximize their profit and pay their credit back. Then to satisfy the owners’ interest, drivers drive with speed and overload passengers…“(Driver)*

### Theme 4: Financial and technological issues

The majority of drivers shared the idea that the current tariff is not proper and adequate, even to cover drivers’ assistance allowance and buy spare parts. They explained that they engaged in RDBs like speeding and overloading passengers to maximize their profit. Most participants also frequently mentioned that many vehicles have major technical problems. These technical problems include the absence of a seat belt, rear and side mirrors, and indicator lights.

“Unless *you overload passengers, you will not be able to cover the driver’s allowance and maximize the amount of money given to the owner of the car “(Driver*).

“*Overloading is because of the highest cost of spare parts, only having 24 passengers (the allowed number of passengers) may end up in bankruptcy.“ (Driver)*

Most of the participants perceived that the reason behind the technical problems of vehicles was related to either the owner of the car, who delays the maintenance of the vehicle, or the transport offices, which have the mandate to check and certify the technical competency of vehicles. Most of the participants here brought up road and transport office staff problems as a big concern. Most participants stated that, even though cars are obliged to undergo annual technical examinations at the road and transport office, they are giving certificates even for cars that didn’t come to the site of the examination. They also indicated the gap in following and correcting vehicles with major technical defects. They described how, as a technical examination is a one-day checkup, owners could borrow some parts of the car from other vehicles, and after getting the license, they could turn it back over to the owner. All the aforementioned technical problems of the vehicle were considered a major cause for drivers’ engagement in RDBs, like turning without showing a light signal, driving without a seat belt, and driving without checking the side view mirror, which could contribute to the occurrence of accidents.

*“Because of poor periodic technical checkups, we found cars working devoid of a seat belt, a back mirror, a functional indicator light, and even with a cracked windshield. All these technical problems on the vehicle oblige the driver to drive without a seat belt, to turn without showing a light... (Traffic Police Officer)*

*”Since technical checkups are conducted annually, one can borrow some part of the vehicle from his friend and give it back after getting the competence certificate…“(Driver)*

## Discussion

The main purpose of this study was to explore perceptions related to drivers’ engagement in RDBs. Four themes were identified, such as training curriculum and application gaps, transport safety law and implementation problems, passengers’ and car owners’ related problems, and financial and technical problems.

According to this study, the driver training curriculum had gaps in several areas, including the adequacy of training duration, the clarity of the training manual, and the quality of the exit exam. Furthermore, there were implementation flaws such as admitting trainees based on predetermined entry criteria, delivering training based on curriculum, and licensing only passed trainees. This finding is also supported by the study conducted in Iran to explore antisocial driving behavior, which reported that driver trainees who received inadequate specialized training could not bring about behavior change [22]. Since the entry of younger, less experienced [22–24], and poorly trained drivers into the profession increases the risk of RTCs [22], proper and standardized methods of professional drivers’ qualification, certification, and control of working hours should be in place, as already recommended by the United Nations (UN) Assembly [25].

Globally, 123 countries have laws for at least one of the five major RDBs (speed, driving drunk, seat belt use, helmet use, and child restraint); however, the gap in reinforcing the law remains challenging [2]. This study also reported that road safety law enforcement, which includes the nature of road safety rules and regulations and the level of enforcement, was among the driving forces for RDBs. This finding is supported by different studies done in Iran [22, 26] that emphasized the vital role of governance and long-term rule enforcement to create societal values that could abhor and prevent risky driving spontaneously [27]. Exaggerated law enforcement measures were also mentioned as contributing issues for RDBs in this study. When drivers were punished with higher level measures as compared to committed violations, they could get emotional and get out of control of the car. This finding is also supported by another systematic review, which showed that drivers with punishment may get emotional and be poor at concentrating on their driving tasks [28]. In this study, violation of one or more rules was identified as one motivator for engaging in other risky behaviors. This finding is also supported by evidence from a study done in Italy, which reported that reckless drivers were more likely to engage in multiple, related RDBs [29]. Thus, endorsement and implementation of inclusive road safety legislation have a pivotal role in reducing RTCs [25].

According to the findings of this study, passengers, especially those who take the front seat, can influence the behavior of the driver, and this result is supported by a survey done in the United States of America that showed drivers increase their speed when they go with passengers, especially with their friends, compared to when driving alone [30]. Some participants also frequently mentioned that when peers and females take front passenger seats, the drivers’ engagement in RDBs increases. This finding is different from other studies, which reported that when females are involved as passengers, drivers’ involvement in RBBs is lower as compared with male passengers [31–33]. The difference could be due to the difference in socio-cultural differences in study participants since studies used for comparison are from developed countries, but here a quantitative study is needed to confirm and quantify the size of the influence of having peer and female passengers on the behavior of drivers in the study area. This implies that educating the community based on current scientific evidence could bring about change in reducing RDB [25, 32, 33]. In addition, this study also indicated the influence of vehicle owners’ perceptions on drivers’ behavior. They influence drivers’ behavior by pushing them to overload passengers and drive at speed. In addition, they could also hire less skilled and experienced drivers to decrease their expenses and maximize their income. Furthermore, owners were also key people in solving technical problems related to the vehicle. As the evidence confirmed, poor maintenance and the absence of regular service are the major causes of RTCs [27]. So, behavior-change communications focusing on owners of the car could bring significant benefits.

The result of this study also emphasized the role financial and technical issues play in drivers’ engagement in RDB. Even though drivers have adequate operating skills based on road safety laws and regulations, they may not be able to do so if the vehicle system is not functioning well. This finding is also supported by a study conducted in the United Arab Emirates [26] that reported that the absence of regular technical checkups of vehicles is a major cause of RDBs that end up with devastating RTCs. Thus, as directed by the UN Assembly on Road Safety to reduce RTCs, there should be periodic and standardized checkups for old and newly entered vehicles [25]. Furthermore, this study also found that drivers need more time to earn more money, and because of this, they cannot give priority to passengers and other vehicles; they can improperly overtake and drive beyond the speed limit. This result is also supported by a narrative review done in Iran [27]. As a result, early implementation of educational interventions and road safety law enforcement could be beneficial in breaking the vicious cycle formed by each RDB, such as driving drunk, speeding, and overloading.

## The strength and limitations of the study

This study applied qualitative methods to generate essential evidence about contributing issues to drivers’ engagement in RDBs, which is easily modifiable as compared to improving the quality of vehicles and road infrastructure in resource-limited countries like Ethiopia, and a major cause of RTCs. This study is not entirely limitless. The study was carried out in one city only, which may miss some details of other areas. It also focuses on public transport vehicle drivers and excludes other vehicle drivers. Moreover, this study could generate more important evidence if it applied a mixed approach.

## Conclusion

This study found gaps in drivers’ training curriculum and its implementation and problems related to road safety law and enforcement as the driving forces behind drivers’ engagement in RDBs. Furthermore, problems related to passengers’ and car owners’ behavior and technical and financial problems were also the main contributing issues to RDBs. Hence, revision and proper implementation of drivers’ training curriculum, transport tariffs, and road safety laws with sound strategic behavior change communication tailored to passengers’ and car owners’ behavior could be major strategies to reduce RDBs.

## Electronic supplementary material

Below is the link to the electronic supplementary material.

Supplementary Material 1

## Data Availability

The data used during the current study are available from the corresponding author upon request.

## Abbreviations

RDBs: Risky Driving Behaviors
RTCs: Road Traffic Crashes
UN: United Nations
