# Supplementary material for: A qualitative study of perception related to risky driving behavior in Debre Markos City, North West Ethiopia, 2021
Source: BMC Public Health. 2023 May 26;23:977. doi: 10.1186/s12889-023-15862-x (PMC10223845; doi:10.1186/s12889-023-15862-x)
Supplement: Supplementary file 1 — Supplementary Material 1 [file 12889_2023_15862_MOESM1_ESM.docx]

## English version interview guide

Interview guide prepared to be used in interviewing public transport vehicle drivers to explore pushing factors of drivers to RDB.

Well, come.

My name is Elyas Melaku, and I come from the University of Gondar to collect data for a study done on pushing factors of risky driving behavior among passenger vehicle drivers in Debre Markos City, in 2021

This study aims to understand pushing factors that increase driver engagement in RDB, so the data that you will give me is very important to health promotion activities at local, regional, and national levels. In this study, we will talk about pushing factors that increase East Gojjam zone public transport drivers’ engagement in RDB.

Any information that you will share with me during the interview will help professionals, researchers, and policymakers to perceive the problem well.

Before we proceed to the main interview, here, there are points that you should know.

- We are here to learn from you, and the question that we ask has no right or wrong answer; we need your personal opinion.
- During the interview, you are not expected to call your name or your organizations name.
- If you have any questions that you don’t want to answer for any reason, we can continue to the next question.
- You can stop the interview at any time.
- All information that you will give to us will be kept confidentiality

The interview may take up to 1 hr., but to remind what you say and to make data suitable for the analysis, I will use a voice recorder when we start the interview.

Do you have any questions?

Do you agree to participate?

1. Yes B. No

Part I Socio-demographic data

1. Age
2. Sex
3. Marital status
4. Educational status
5. Driving experience
6. Car type (carrying capacity)
7. Previous involvement in a road traffic accident

Part II pushing factors to risky driving behavior

1. How do you see road traffic crashes occurring in the East Gojjam zone?
2. The magnitude of RTA?
3. From your perspective what are the causes of RTA?
4. Could you explain the consequence of RTA?
5. How often do practice risky driving behavior while you drive (speeding, improper overtaking, driving drunk, overloading passengers, driving after chewing, and making a phone call)?
6. What is the role of drivers’ behavior in the reduction or increment of road traffic crashes? Could you explain it more?
7. What are the pushing factors that increase your engagement in risky driving behavior (speeding, improper overtaking, driving drunk, overloading passengers, driving after chewing, and making a phone call) while you drive?
8. Could you explain issues that could contribute to your engagement in risky driving behavior? (starting from registration at driver training school to the issuance of the license)
9. What is the effect of current road safety laws on your risky driving behavior? (including its implementation)
10. What should be done to reduce your (drivers’) engagement in risky driving behavior?
11. What else do you want to add about pushing factors that increase drivers’ engagement in risky driving behavior?

Thank you!

**Interview guide prepared to be used in interviewing traffic police officers to explore pushing factors of RDB among public transport drivers in East Gojjam zone, North West Ethiopia**

Welcome.

My name is Elyas Melaku, and I come from the University of Gondar to collect data for a study done on risky driving behavior and associated factors

This study aims to understand pushing factors that increase driver engagement in RDB, so the data that you will give me is very important to health promotion activities at the local, regional, and national levels. In this study, we will talk about pushing factors that increase East Gojjam zone public transport drivers’ engagement in RDB.

Any information that you will share with me during the interview will help professionals, researchers, and policymakers to perceive the problem well.

Before we proceed to the main interview, here, there are points that you should know.

- We are here to learn from you, and the question that we ask has no right or wrong answer; we need your personal opinion.
- During the interview, you are not expected to call your name or your organizations name.
- If you have any questions that you don’t want to answer for any reason, we can continue to the next question.
- You can stop the interview at any time.
- All information that you will give us will be kept confidentiality

The interview may take up to 1 hr., but to remind what you say and to make data suitable for the analysis, I will use a voice recorder when we start the interview.

Do you have any questions?

Do you agree to participate?

1. Yes B. No

Part I Socio-demographic data

1. Age
2. Sex
3. Educational status
4. Marital status
5. Occupation
6. work experience

Part II pushing factors to risky driving behavior

1. How do you see road traffic crashes occurring in the East Gojjam zone? its
2. The magnitude of RTA?
3. From your perspective what are the causes of RTA?
4. Could you explain the consequence of RTA?
5. From your experience and exposure, how do you describe the consequence of road traffic accidents caused by drivers’ risky driving behavior?
6. What is the role of drivers’ behavior in the reduction or increase of road traffic crashes? Could you explain it more?
7. What are pushing factors that increase drivers' engagement in risky driving behavior (overspeeding, improper overtaking, driving drunk, overloading passengers, driving after chewing, and making a phone call) while you drive?
8. Could you explain issues that could contribute to drivers' engagement in risky driving behavior? (starting from registration at driver training school to the issuance of s license)
9. What is the effect of current road safety laws on drivers' risky driving behavior? (including its implementation)
10. What should be done to reduce drivers’ engagement in risky driving behavior?
11. What else do you want to add about pushing factors that increase drivers’ engagement in risky driving behavior?

**Interview guide prepared to be used in interviewing driver training school instructors to explore pushing factors of RDB among public transport drivers in East Gojjam zone, North West Ethiopia**

Welcome.

My name is Elyas Melaku, and I come from the University of Gondar to collect data for a study done on risky driving behavior and associated factors

This study aims to understand well-pushing factors that increase driver engagement in RDB, so the data that you will give me is very important to health promotion activities at the local, regional, and national levels. In this study, we will talk about pushing factors that increase East Gojjam zone public transport drivers’ engagement in RDB.

Any information that you will share with me during the interview will help professionals, researchers, and policymakers to perceive the problem well.

Before we proceed to the main interview, here, there are points that you should know.

- We are here to learn from you, and the question that we ask has no right or wrong answer; we need your personal opinion.
- During the interview, you are not expected to call your name or your organizations name.
- If you have any questions that you don’t want to answer to for any reason, we can continue to the next question.
- You can stop the interview at any time.
- All information that will give to us will be kept confidentiality

The interview may take up to 1 hr., but to remind what you say and to make data suitable for the analysis, I will use a voice recorder when we start an interview.

Do you have any questions?

Do you agree to participate?

1. Yes B. No

Part I Socio-demographic data

1. Age
2. Sex
3. Marital status
4. Educational status
5. Occupation
6. work experience

Part II pushing factors to risky driving behavior

1. How do you see road traffic crashes occurring in the East Gojjam zone?
2. The magnitude of RTA?
3. From your perspective, what are the causes of RTA?
4. Could you explain the consequence of RTA?
5. What is the role of drivers’ behavior in the reduction or increase of road traffic crashes? Could you explain it more?
6. What are the pushing factors that increase drivers' engagement in risky driving behavior (overspeeding, improper overtaking, driving drunk, overloading passengers, driving after chewing, and making a phone call) while you drive?
7. What is the role of driving school instructors regarding drivers' risky driving behavior? (measures taken by instructors)?
8. What is the effect of the current driver training curriculum on drivers’ risky driving behavior?
9. Could you explain issues that could contribute to drivers' engagement in risky driving behavior? (starting from registration at school to issuance of the s license)
10. What else do you want to add about pushing factors that increase drivers’ engagement in risky driving behavior?
